# Mn Doping-Induced Charge-Carrier Redistribution in Co_3_O_4_ for Enhanced CO_2_ Photoreduction Toward CH_4_ with H_2_O

**DOI:** 10.3390/molecules31173120

**Published:** 2026-09-06

**Authors:** Gaofeng Zhou, Wenchao Shangguan, Xuan Wang, Suhang Wang, Kaiyun Li, Shiqing Li, Ying Ma, Sugang Meng, Shifu Chen

**Affiliations:** 1 Key Laboratory of Green and Precise Synthetic Chemistry and Applications, Ministry of Education, School of Chemistry and Chemical Engineering, Huaibei Normal University, Huaibei 235000, China; f3186329479@163.com (G.Z.);; 2 Anhui Provincial Key Laboratory of Synthetic Chemistry and Applications, School of Chemistry and Chemical Engineering, Huaibei Normal University, Huaibei 235000, China; 3 School of Life Sciences, Huaibei Normal University, Huaibei 235000, China

**Keywords:** Co_3_O_4_, Mn doping, charge-carrier separation, photocatalytic CO_2_ methanation, in situ spectroscopy

## Abstract

Photocatalytic CO_2_ reduction to CH_4_ with H_2_O is hindered by rapid charge recombination and sluggish multielectron/proton-coupled hydrogenation kinetics. Herein, we show that Mn doping induces charge-carrier redistribution within Co_3_O_4_, thereby enhancing CO_2_ photoreduction to CH_4_ under sacrificial-agent-free conditions. The optimized Mn5–Co_3_O_4_ achieves CH_4_ and CO production rates of 16.9 and 9.8 μmol g^−1^ h^−1^, respectively, with its CH_4_ production rate reaching 10.6 times that of pristine Co_3_O_4_. Mechanistic investigations indicate that Mn doping modulates carrier dynamics and surface-intermediate hydrogenation. Photoelectrochemical and photoluminescence measurements demonstrate that Mn incorporation promotes charge-carrier separation, with Mn5–Co_3_O_4_ exhibiting the most favorable separation efficiency, as reflected in an extended average photoluminescence lifetime of 23.17 ns compared with 9.95 ns for pristine Co_3_O_4_. In situ irradiated X-ray photoelectron spectroscopy shows shifts of the Mn and Co signals toward lower and higher binding energies, respectively, indicating electron enrichment at Mn sites and hole accumulation at Co sites. In situ Fourier-transform infrared spectroscopy further reveals enhanced bands tentatively assigned to *COOH, *CHO, and *CH_3_O intermediates, supporting their progressive hydrogenation toward CH_4_. These findings provide a mechanistic framework for coordinating charge redistribution with surface hydrogenation during multielectron/proton-coupled CO_2_ conversion with H_2_O.

## 1. Introduction

Solar-driven photocatalytic and photoelectrochemical CO_2_ reduction offers a promising route for carbon utilization and solar-to-chemical energy conversion [1,2,3,4,5,6,7,8]. Replacing organic sacrificial agents with H_2_O as the proton source simplifies the reaction system and more closely emulates the redox framework of artificial photosynthesis [9,10,11,12,13,14,15]. Pairing CO_2_ reduction with value-added organic oxidation can improve photogenerated-carrier utilization [16,17,18], but such systems rely on organic substrates and do not address the kinetic constraints of CO_2_/H_2_O artificial photosynthesis. Among accessible carbon products, CH_4_ is particularly attractive owing to its high energy density and compatibility with existing energy infrastructure. Recent photothermal methanation studies further underscore the importance of regulating hydrogenation-active interfaces to favor CH_4_ formation [19]. However, CH_4_ formation requires the sequential transfer of eight electrons and eight protons to a single carbon center, rendering the overall process kinetically demanding. Moreover, the competing CO evolution and hydrogen evolution reaction (HER) further constrain selectivity toward CH_4_ [20,21,22]. These limitations arise from two bottlenecks. Rapid electron–hole recombination reduces the number of photogenerated carriers reaching the catalyst surface, whereas sluggish protonation kinetics can arrest carbon reduction at less-reduced products such as CO [23,24]. Therefore, coordinating these two processes within a single photocatalyst remains a challenge in achieving efficient CO_2_ methanation.

Co_3_O_4_ has attracted considerable attention for photocatalytic CO_2_ reduction and methanation, owing to its broad optical absorption, multiple accessible Co oxidation states, and surface chemistry favorable for CO_2_ adsorption and activation [25,26,27]. To date, strategies such as oxygen-vacancy engineering, heterojunction construction, cocatalyst loading, and compositional modification have been developed to optimize individual steps within the photocatalytic process [25,28,29,30,31,32,33,34,35,36,37]. However, most of these strategies target CO_2_ capture, activation, or charge-carrier separation, whereas the conversion of CO_2_ to CH_4_ additionally demands sustained hydrogenation. Deep reduction requires the continuous generation and transformation of hydrogenated intermediates, including *CHO and *CH_3_O, through stepwise reactions on the same adsorbed carbon species. Studies of CO_2_ hydrogenation likewise show that the availability and transfer of surface hydrogen species can govern downstream conversion [38]. Critically, these reaction steps must be completed before the reductive carriers are dissipated through recombination or diverted to the competing hydrogen evolution reaction [23,25]. Consequently, improving CO_2_ adsorption or narrowing the band gap alone does not guarantee efficient CH_4_ production, and a strategy that simultaneously regulates bulk carrier dynamics and surface hydrogenation on Co_3_O_4_ is required.

Heteroatom doping offers a viable strategy to achieve this goal, as it modifies the lattice environment, local electron density, and carrier dynamics within a single oxide framework. Among candidate dopants, manganese (Mn) is particularly well suited to tuning the local charge environment of Co_3_O_4_, owing to its multivalent nature and strong electronic interaction with the Co–O framework. Building on this rationale, Mn has been introduced into Co_3_O_4_ to construct adjacent Mn–Co redox-active sites that promote H_2_O activation and CO_2_ methanation [23]. Atomic Mn incorporation has also been reported to regulate charge transfer and enhance CO evolution under dilute-CO_2_ and pure-water conditions [39]. These studies confirm that Mn doping modifies both the electronic properties and the surface reactivity of Co_3_O_4_ [40]. However, the relationships among the Mn doping level, carrier lifetime, and deep hydrogenation remain unclear. Therefore, how to simultaneously ensure a sufficiently long effective lifetime of photogenerated carriers and achieve efficient consecutive hydrogenation of adsorbed carbon intermediates through an appropriate Mn doping level, thereby converting carbon products into CH_4_, is a scientific issue that urgently needs to be addressed.

Inspired by these considerations, we demonstrate that incorporating an appropriate level of Mn into Co_3_O_4_ effectively couples charge-carrier regulation with the stepwise hydrogenation of CO_2_-derived intermediates, thereby enabling sacrificial-agent-free photocatalytic reduction of CO_2_ and H_2_O to CH_4_. The optimized Mn5–Co_3_O_4_ catalyst achieves CH_4_ and CO production rates of 16.9 and 9.8 μmol g^−1^ h^−1^, respectively, representing 10.6-fold and 4.1-fold enhancements over pristine Co_3_O_4_, and raises the CH_4_/CO molar ratio from 0.67 to 1.72. Mechanistic investigations reveal that photogenerated electrons preferentially accumulate at Mn sites, whereas holes remain localized within the Co_3_O_4_ framework; this spatial charge separation suppresses electron–hole recombination and extends the average photoluminescence lifetime from 9.95 ns to 23.17 ns. Time-resolved in situ Fourier-transform infrared spectroscopy further reveals that Mn5–Co_3_O_4_ sustains continuous accumulation of *COOH, *CHO, and *CH_3_O intermediates, thereby enabling efficient CH_4_ generation.

## 2. Results and Discussion

### 2.1. Synthesis and Structural Characterization

**Scheme 1:**
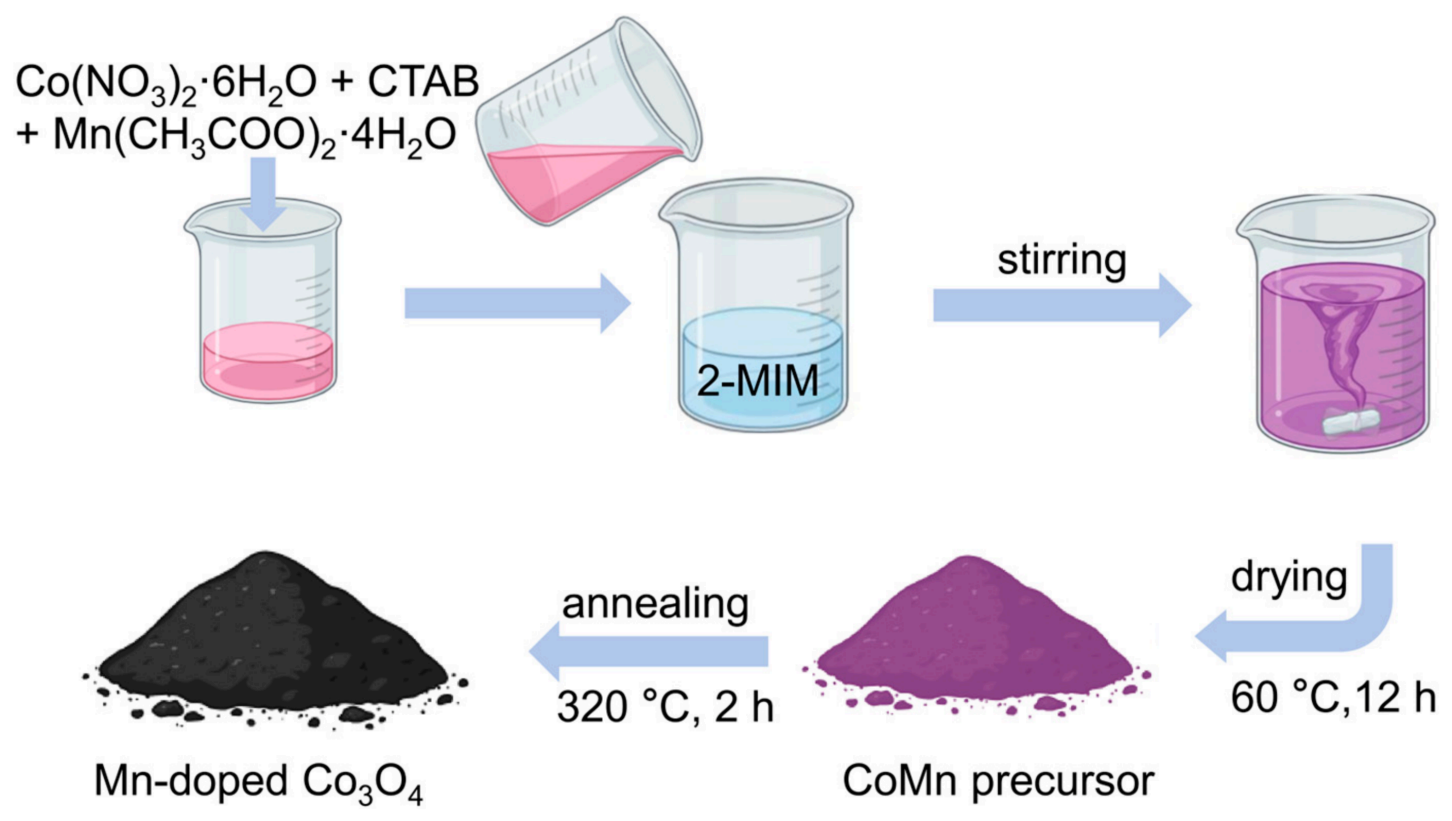
Preparation of the Mn-doped Co_3_O_4_ photocatalyst through precursor co-assembly and thermal conversion.

The Mn-doped Co_3_O_4_ series was prepared by precursor co-assembly followed by thermal conversion (Scheme 1). Co(NO_3_)_2_·6H_2_O and different amounts of Mn(CH_3_COO)_2_·4H_2_O were co-dissolved in a CTAB-containing aqueous solution, before the resulting mixture was rapidly introduced into an aqueous 2-methylimidazole (2-MIM) solution. The coordination of Co^2+^ and Mn^2+^ with 2-MIM afforded a Co–Mn-containing precursor in which both metal species were co-assembled before thermal conversion. Subsequent thermal conversion in air at 320 °C yielded Mn-modified Co_3_O_4_-type oxides. The obtained samples were labeled Mn5–Co_3_O_4_, Mn10–Co_3_O_4_, and Mn20–Co_3_O_4_, where 5, 10, and 20 denote the mass (in mg) of Mn(CH_3_COO)_2_·4H_2_O introduced during synthesis rather than the Mn content of the final solids.

**Figure 1:**
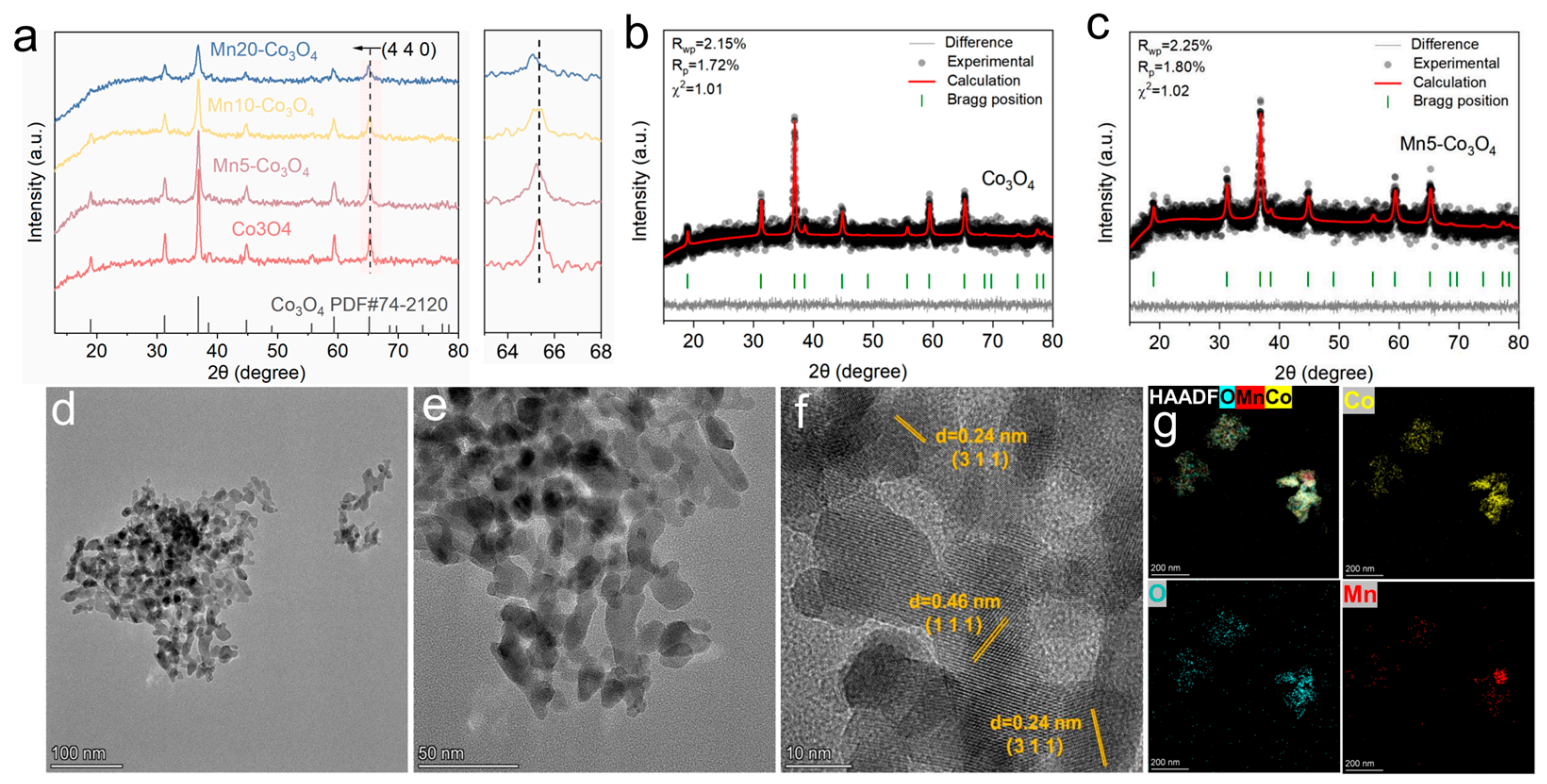
(**a**) XRD patterns of pristine Co_3_O_4_ and the Mn5–Co_3_O_4_, Mn10–Co_3_O_4_, and Mn20–Co_3_O_4_ samples; the inset shows the 63–68° region containing the (440) reflection. Rietveld refinement profiles of (**b**) Co_3_O_4_ and (**c**) Mn5–Co_3_O_4_. (**d**,**e**) Representative TEM and (**f**) HRTEM images of Mn5–Co_3_O_4_. (**g**) HAADF-STEM image and corresponding elemental maps of Co, O, and Mn acquired from the same region.

As shown in Figure 1a, all samples exhibited diffraction peaks at approximately 19.0°, 31.3°, 36.8°, 38.5°, 44.8°, 49.0°, 55.7°, 59.4°, and 65.2°, which were indexed to the (111), (220), (311), (222), (400), (331), (422), (511), and (440) planes of cubic Co_3_O_4_ (JCPDS #74-2120) [41]. No additional Bragg reflections attributable to crystalline MnO_x_ phases were detected across the Mn dosage series. The (440) reflection shifted progressively toward lower angles from 65.23° for pristine Co_3_O_4_, corresponding to an increase in the (440) interplanar spacing and supporting structural modification of the Co_3_O_4_ lattice following Mn incorporation. To obtain further structural information, Rietveld refinements of the pristine Co_3_O_4_ and Mn5–Co_3_O_4_ diffraction patterns were performed using a cubic spinel structural model with the Fd−3m space group (Figure 1b,c). The calculated profiles closely reproduced the experimental diffraction patterns. The refined lattice parameter increased from 8.0799(7) Å for Co_3_O_4_ to 8.0847(12) Å for Mn5–Co_3_O_4_, while the corresponding unit-cell volume increased from 527.50(14) to 528.44(23) Å^3^. For Mn5–Co_3_O_4_, the diffraction pattern was satisfactorily described by a structural model in which Mn occupies the octahedral 16d site together with Co, whereas the tetrahedral 8a site remains occupied by Co. These results support an octahedral-site structural model for Mn incorporation into the Co_3_O_4_-type spinel framework. The refinements yielded R_p_/R_wp_/χ^2^ values of 1.72%/2.15%/1.01 for Co_3_O_4_ and 1.80%/2.25%/1.02 for Mn5–Co_3_O_4_, supporting the reliability of the fitted structural models. The corresponding crystallographic parameters are summarized in Table 1 and Table 2.

ICP-OES measurements showed that the Mn contents of Mn5–Co_3_O_4_, Mn10–Co_3_O_4_, and Mn20–Co_3_O_4_ were 2.87, 6.22, and 13.01 wt.%, respectively, while the corresponding Co contents were 70.93, 67.87, and 60.86 wt.%. These values yielded Mn/Co molar ratios of 0.0435, 0.0983, and 0.2293, respectively. When normalized to three total metal cations under the Co_3_O_4_-type spinel convention, the corresponding cation compositions were Co_2.875_Mn_0.125_O_4_, Co_2.731_Mn_0.269_O_4_, and Co_2.440_Mn_0.560_O_4_. These formulas represent cation-normalized bulk compositions because the oxygen stoichiometry was not independently determined.

TEM images revealed that Mn5–Co_3_O_4_ consisted of interconnected nanoparticles (Figure 1d,e). The HRTEM image in Figure 1f displayed well-resolved lattice fringes with spacings of 0.24 and 0.46 nm, corresponding to the (311) and (111) planes of spinel Co_3_O_4_, respectively [40,42]. No additional lattice fringes attributable to crystalline MnO_x_ or a continuous amorphous shell were observed within the examined region. HAADF-STEM imaging and the corresponding EDS maps showed the co-distribution of Co, O, and Mn within the same particle aggregate, with no obvious Mn enrichment at the particle boundaries (Figure 1g). Together, the diffraction and microscopy results support Mn incorporation into, or close association with, the Co_3_O_4_-type framework. Nevertheless, conventional PXRD and local electron-microscopy measurements cannot quantify or completely exclude a minor X-ray-amorphous, highly dispersed, or nanocrystalline Mn-containing component.

**Figure 2:**
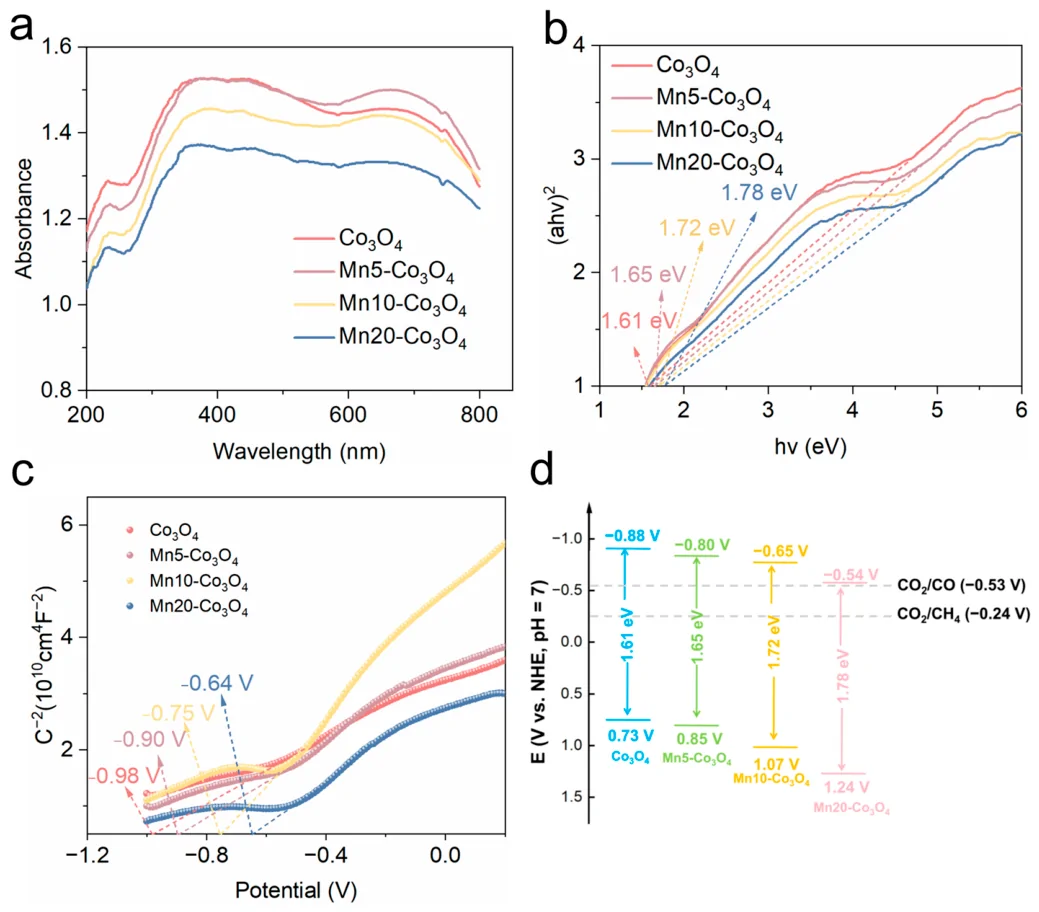
(**a**) UV–visible absorption spectra, (**b**) direct-allowed Tauc plots of the lower-energy apparent optical transition, (**c**) Mott–Schottky plots, and (**d**) schematic band-edge diagram of pristine Co_3_O_4_ and the Mn-modified Co_3_O_4_ samples.

The optical response of the photocatalysts was first examined by UV–vis diffuse reflectance spectroscopy. As shown in Figure 2a, all samples exhibit broad absorption across the 200–800 nm range, encompassing the emission spectrum of the xenon lamp used in the photocatalytic tests [41]. Among the Mn-containing samples, Mn5–Co_3_O_4_ retained an absorption intensity comparable to that of pristine Co_3_O_4_ across most of the visible region, whereas the absorption intensity decreased progressively at higher Mn precursor dosages. Following the direct-allowed Tauc construction commonly applied to spinel Co_3_O_4_ and Mn-modified Co_3_O_4_ [39,41,43], the lower-energy apparent optical transition energies were estimated to be 1.61, 1.65, 1.72, and 1.78 eV for pristine Co_3_O_4_, Mn5–Co_3_O_4_, Mn10–Co_3_O_4_, and Mn20–Co_3_O_4_, respectively. Small variations in the lower-energy optical transition have also been reported for Mn-doped Co_3_O_4_ materials [44]. Because Co_3_O_4_ exhibits multiple optical transitions and the extrapolated intercept depends on the selected fitting interval, these values should be regarded as approximate apparent transition energies rather than statistically resolved differences in the intrinsic band gap [45]. Overall, the samples retain the broad visible-light absorption characteristics of the Co_3_O_4_ host, and the photocatalytic activity trend is discussed primarily in relation to charge-carrier dynamics rather than the small differences among the Tauc-derived values.

Mott–Schottky measurements were then performed to estimate the band-edge positions. All samples displayed positive slopes (Figure 2c), consistent with n-type semiconductor behavior [46]. The flat-band potentials extrapolated from the linear regions were −0.98, −0.90, −0.75, and −0.64 V versus Ag/AgCl for Co_3_O_4_, Mn5–Co_3_O_4_, Mn10–Co_3_O_4_, and Mn20–Co_3_O_4_, respectively. Converting these values to the NHE scale (*E*_NHE_ = *E*_Ag/AgCl_ + 0.197 V) and taking the conduction band minimum of an n-type semiconductor to be 0.1 V more negative than the flat-band potential [46] gave conduction-band positions of −0.88, −0.80, −0.65, and −0.54 V. The progressive positive shift of the Mott–Schottky-derived flat-band potential with increasing Mn precursor dosage indicates a composition-dependent modification of the equilibrium electronic structure. Previous studies of Mn_x_Co_3−x_O_4_ spinels have shown that the Mn/Co composition and cation-site occupation can regulate Mn/Co 3d–O 2p hybridization, Mn-associated electronic states near the Fermi level, and mixed-valence charge transport [39,47,48,49]. In the present series, the systematic shift of the (440) reflection and associated lattice expansion, together with the Co 2p and lattice O 1s shifts observed for Mn5–Co_3_O_4_ relative to pristine Co_3_O_4_, are consistent with structural and electronic perturbation of the Co–O framework. Accordingly, the progressively less-negative apparent conduction-band positions are interpreted as reflecting a positive shift in the apparent flat-band potential associated with Mn-induced modification of the Co–O–Mn electronic structure. Because Mott–Schottky analysis does not resolve individual orbital contributions, this explanation is presented as a plausible interpretation rather than a unique microscopic mechanism. Combining the apparent conduction-band positions with the corresponding lower-energy apparent optical transition energies gave estimated valence-band positions of 0.73, 0.85, 1.07, and 1.24 V, respectively. The estimated band-edge positions indicate that the Mn-containing samples retain potentials compatible with the relevant CO_2_-reduction and H_2_O-oxidation processes under the tested conditions (Figure 2d). However, because the optical-transition energies and Mott–Schottky-derived positions are operational estimates, the relatively small differences among adjacent compositions should not be used alone to explain the pronounced photocatalytic activity trend. The band analysis is therefore used to assess thermodynamic compatibility, whereas the composition-dependent activity is interpreted primarily in relation to the carrier-dynamics and interfacial-reaction evidence discussed below.

### 2.2. Photocatalytic CO_2_ Reduction Performance

**Figure 3:**
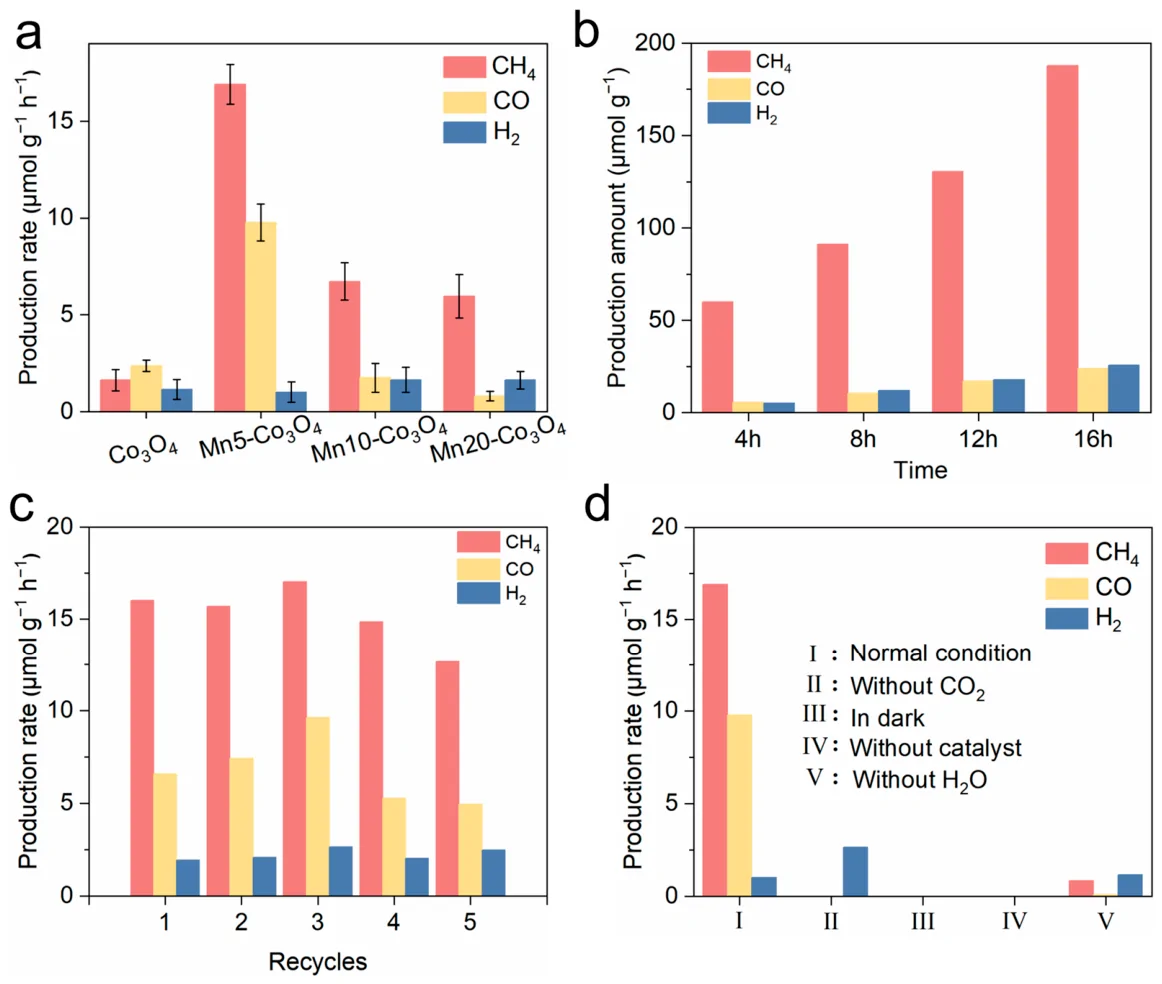
(**a**) CH_4_, CO, and H_2_ production rates over Co_3_O_4_, Mn5–Co_3_O_4_, Mn10–Co_3_O_4_, and Mn20–Co_3_O_4_. The values are means from three independent photocatalytic experiments, and the error bars represent the corresponding standard deviations (n = 3). (**b**) Cumulative CH_4_, CO, and H_2_ amounts over Mn5–Co_3_O_4_ during a continuous 16 h irradiation experiment. (**c**) Cycling performance of Mn5–Co_3_O_4_ over five consecutive cycles. (**d**) Control experiments under the complete reaction conditions and in the absence of CO_2_, light, photocatalyst, or H_2_O.

Photocatalytic CO_2_ reduction was carried out without any sacrificial agent, using 20 mg of photocatalyst and 2 mL of H_2_O under full-spectrum irradiation from a 300 W Xe lamp at 25 °C. As shown in Figure 3a, pristine Co_3_O_4_ delivered CH_4_ and CO production rates of only 1.6 and 2.4 µmol g^−1^ h^−1^, respectively. This limited activity is consistent with the band alignment established above, as the valence band of Co_3_O_4_ was less positive than the H_2_O/O_2_ potential and photogenerated holes could therefore not be consumed efficiently by water oxidation in a sacrificial-agent-free system. Mn doping substantially enhanced the activity, and the performance followed a volcano-shaped dependence on the Mn dosage. Mn5–Co_3_O_4_ exhibited the highest activity, with CH_4_ and CO production rates of 16.9 and 9.8 µmol g^−1^ h^−1^, corresponding to 10.6- and 4.1-fold increases relative to pristine Co_3_O_4_, respectively, whereas further increasing the Mn dosage lowered the CH_4_ rate to 6.7 µmol g^−1^ h^−1^ for Mn10–Co_3_O_4_ and 6.0 µmol g^−1^ h^−1^ for Mn20–Co_3_O_4_. H_2_ was simultaneously detected over all samples, reflecting the competition between proton reduction and CO_2_ reduction for photogenerated electrons. The CH_4_/CO molar ratio increased from 0.67 over Co_3_O_4_ to 1.72 over Mn5–Co_3_O_4_ and the electron-based CH_4_ fraction among the carbon-containing products increased from 73% to 87%. Thus, among the carbon products, Mn doping increased both the overall CO_2_ conversion and the relative contribution of the eight-electron methanation pathway.

The apparent flat-band potentials shifted progressively toward more positive values with increasing Mn precursor dosage. However, the energetic differences between adjacent compositions were modest, whereas the CH_4_ production rate of Mn5–Co_3_O_4_ was approximately 2.5 times that of Mn10–Co_3_O_4_. Thus, the estimated band positions establish that the relevant redox reactions remain thermodynamically accessible but do not by themselves account for the volcano-shaped activity trend. Additional composition-dependent factors, particularly charge-carrier separation and lifetime, therefore govern the observed activity differences, as discussed below.

The durability of Mn5–Co_3_O_4_ was subsequently assessed. As shown in Figure 3b, the accumulated amounts of CH_4_, CO, and H_2_ increased continuously during 16 h of uninterrupted irradiation, with CH_4_ reaching 188 µmol g^−1^, confirming that the photocatalyst remained active throughout the test. Mn5–Co_3_O_4_ also maintained product formation over five consecutive cycles (Figure 3c). The CH_4_ production rate decreased from approximately 16.0 µmol g^−1^ h^−1^ in the first cycle to approximately 12.6 µmol g^−1^ h^−1^ in the fifth cycle, corresponding to an activity retention of approximately 79%. These experimental results demonstrate that Mn5–Co_3_O_4_ exhibits both enhanced CH_4_ yield and long-term stability.

Control experiments were performed to identify the origin of the products (Figure 3d). No appreciable CH_4_ or CO was detected in the absence of CO_2_, whereas H_2_ was still observed because proton reduction proceeded as a competing reaction. Neither carbon product was detected in the dark or without the photocatalyst, confirming that CH_4_ and CO formation required the simultaneous presence of CO_2_, light, and the photocatalyst. Furthermore, omitting externally added H_2_O markedly suppressed all three products, and the small residual signals are ascribed to water weakly adsorbed on the photocatalyst surface [9]. These results collectively demonstrate that both gaseous CO_2_ and H_2_O participated in the photocatalytic reaction.

### 2.3. Mechanism of Photocatalytic Activity Enhancement

**Figure 4:**
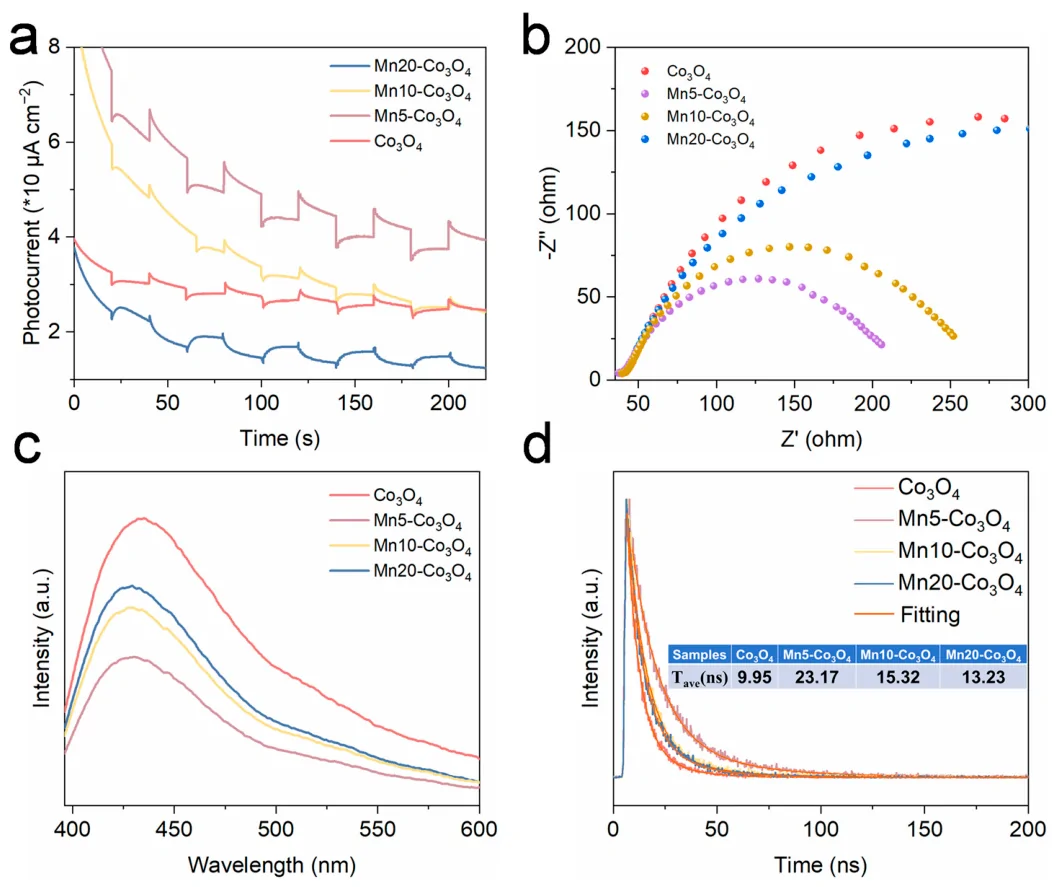
(**a**) Transient photocurrent responses, (**b**) EIS Nyquist plots, (**c**) steady-state PL spectra, and (**d**) TRPL decay curves of Co_3_O_4_, Mn5–Co_3_O_4_, Mn10–Co_3_O_4_, and Mn20–Co_3_O_4_.

Photoelectrochemical measurements were performed to evaluate how Mn dosage affects the utilization of photogenerated charge carriers [33,50]. As shown in Figure 4a, all samples exhibited reproducible photocurrent responses under irradiation, and Mn5–Co_3_O_4_ showed the strongest response, indicating that a larger fraction of photogenerated carriers was separated and transported to the surface. Consistently, Mn5–Co_3_O_4_ displayed the smallest Nyquist arc in the EIS spectra (Figure 4b), reflecting the lowest interfacial charge transfer resistance among the series. Both measurements exhibited the same volcano-shaped dependence on Mn dosage as the photocatalytic activity, whereas samples prepared with higher Mn dosages showed weaker photocurrents and larger arc radii than Mn5–Co_3_O_4_. Subsequently, steady-state and time-resolved photoluminescence spectra were recorded to probe carrier recombination. As shown in Figure 4c, pristine Co_3_O_4_ exhibited the strongest PL signal, which decreased upon Mn doping, with Mn5–Co_3_O_4_ exhibiting the lowest emission intensity. This trend indicates that an appropriate level of Mn doping effectively suppresses radiative electron-hole recombination. The TRPL decay curves in Figure 4d further support this interpretation. Specifically, the average PL lifetimes of Co_3_O_4_, Mn5–Co_3_O_4_, Mn10–Co_3_O_4_, and Mn20–Co_3_O_4_ are 9.95, 23.17, 15.32, and 13.23 ns, respectively, with Mn5–Co_3_O_4_ exhibiting a lifetime 2.33 times that of pristine Co_3_O_4_. This extended lifetime suggests that photogenerated carriers have more time to reach the surface and participate in interfacial redox reactions [26,51].

Recent studies have shown that local coordination environments and spatially organized charge-transfer pathways can regulate photogenerated-carrier localization and transport during photocatalytic CO_2_ reduction [52,53]. More directly, atomic Mn incorporation into Co_3_O_4_ has been reported to accelerate charge-separation dynamics [39]. Together, these studies provide a broader framework for interpreting the composition-dependent photocurrent, EIS, and PL trends observed in the present Mn–Co_3_O_4_ series.

Notably, the trend in average PL lifetime aligns with that of the CH_4_ production rate, both peaking at Mn5–Co_3_O_4_ and declining with further Mn incorporation. This correlation is particularly pronounced for CH_4_ production. The extension in carrier lifetime confers a substantially greater benefit on the CH_4_ pathway than on the CO pathway. As the lifetime increases from 9.95 to 23.17 ns, the CH_4_ production rate rises by 10.6-fold, whereas the CO production rate increases by only 4.1-fold. This disparity reflects the distinct electron demands of the two pathways. Reduction of CO_2_ to CH_4_ proceeds through an eight-electron process that requires multiple sequential electron and proton transfer steps on the same adsorbed carbon intermediate, all of which must be completed before the carrier is lost to recombination. In contrast, reduction to CO requires only two electrons and is far less sensitive to carrier lifetime. Consequently, extending the carrier lifetime confers a disproportionate benefit on the deep hydrogenation pathway, accounting for the observed shift in product selectivity toward CH_4_. Combined with the band-edge analysis discussed above, these results indicate that the superior performance of Mn5–Co_3_O_4_ arises from the combination of sufficient reduction driving force and substantially extended carrier lifetime.

**Figure 5:**
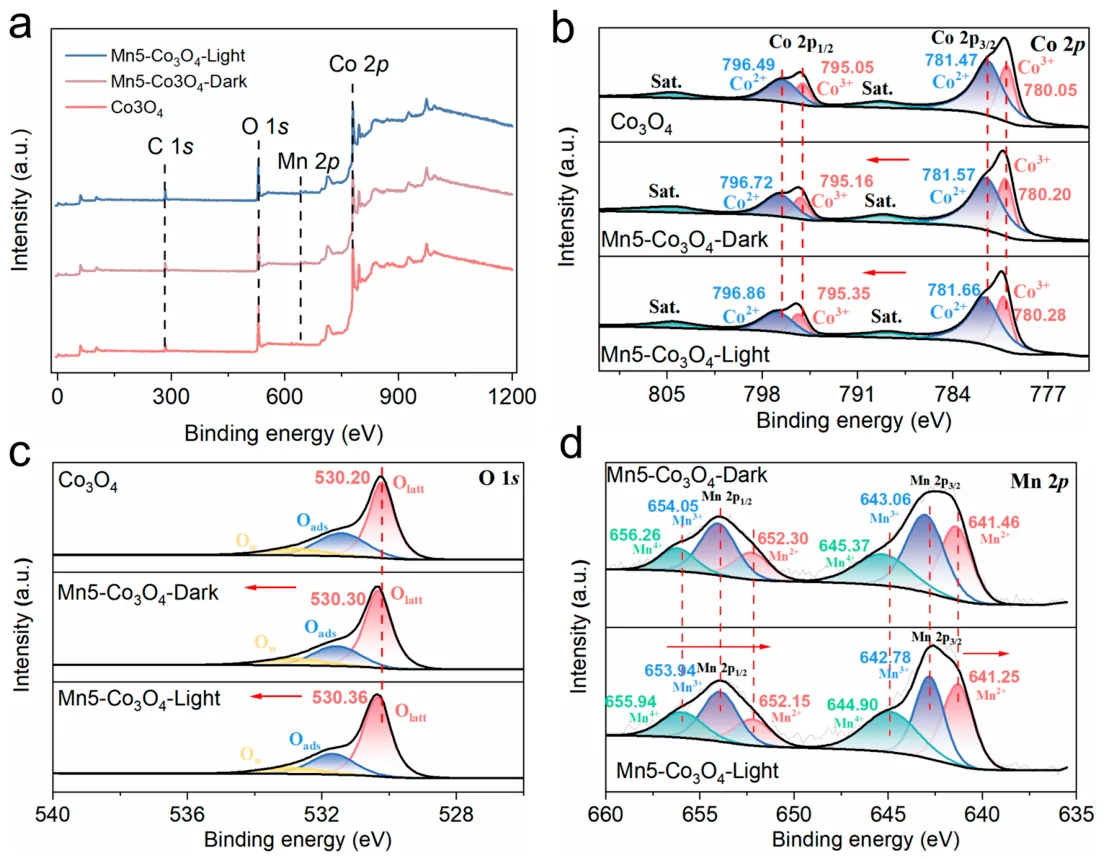
(**a**) XPS survey spectra of pristine Co_3_O_4_ recorded in the dark and Mn5–Co_3_O_4_ recorded in the dark and after 15 min of irradiation. High-resolution (**b**) Co 2p and (**c**) O 1s spectra of pristine Co_3_O_4_ in the dark and Mn5–Co_3_O_4_ in the dark and after 15 min of irradiation using a 300 W Xe lamp. (**d**) Mn 2p spectra of Mn5–Co_3_O_4_ in the dark and after irradiation under the same conditions. The red arrows indicate the direction of the binding energy shifts. All binding energies were calibrated against the C 1s peak at 284.8 eV.

To compare the surface chemical states and examine the light-induced electronic response, XPS measurements were performed on pristine Co_3_O_4_ and the optimized Mn5–Co_3_O_4_ sample. As shown in Figure 5a, the dark-state survey spectra of both samples exhibited C 1s, O 1s, and Co 2p signals. The Mn 2p signal was detected in Mn5–Co_3_O_4_ but was absent, as expected, from pristine Co_3_O_4_, confirming the presence of Mn in the near-surface region of Mn5–Co_3_O_4_. Semi-quantitative analysis of the dark-state XPS survey spectrum gave near-surface atomic concentrations of approximately 41.8, 2.3, and 55.9 at.% for Co, Mn, and O, respectively, after adventitious carbon was excluded from the normalization. The corresponding surface Mn/Co atomic ratio was approximately 0.055, with Mn accounting for approximately 5.2% of the total surface metal cations. When normalized to three metal cations according to the Co_3_O_4_-type spinel convention, this ratio corresponds to an approximate surface cation composition of Co_2.84_Mn_0.16_O_4_. This surface Mn/Co ratio was slightly higher than the bulk value of 0.0435 determined by ICP-OES for Mn5–Co_3_O_4_. Because XPS probes the near-surface region and the O 1s envelope contains contributions from both lattice and surface oxygen species, the XPS-derived value represents a surface cation-normalized composition rather than an exact bulk stoichiometric formula.

The dark-state high-resolution spectra of pristine Co_3_O_4_ and Mn5–Co_3_O_4_ were first compared. Pristine Co_3_O_4_ exhibited Co 2p_3/2_ components at 780.05 eV (Co^3+^) and 781.47 eV (Co^2+^), together with the corresponding Co 2p_1/2_ components at 795.05 eV (Co^3+^) and 796.49 eV (Co^2+^) and two shake-up satellites (Figure 5b) [54]. After Mn incorporation, all Co 2p components shifted to higher binding energies. The lattice-oxygen peak in the O 1s spectrum shifted from 530.20 eV for Co_3_O_4_ to 530.30 eV for Mn5–Co_3_O_4_, whereas the surface-adsorbed oxygen and adsorbed-water components remained at higher binding energies (Figure 5c). The Mn 2p_3/2_ region of Mn5–Co_3_O_4_ was deconvoluted into Mn^2+^, Mn^3+^, and Mn^4+^ components at 641.46, 643.06, and 645.37 eV, respectively (Figure 5d) [39,40], indicating the mixed-valence character of Mn. Because Mn was introduced from an Mn(II) precursor, the XPS components assigned to Mn(III) and Mn(IV) after calcination are attributed primarily to oxidation by molecular oxygen during thermal conversion in air, consistent with the reported formation of mixed-valence manganese oxides from Mn(CH_3_COO)_2_·4H_2_O under oxidative heating conditions [55].

Under irradiation, the Co- and Mn-related signals shifted in opposite directions. The Co 2p_3/2_ components assigned to Co^3+^ and Co^2+^ shifted to higher binding energies of 780.28 and 781.66 eV, respectively, and the O_latt_ peak shifted to 530.36 eV, whereas the Mn^2+^, Mn^3+^, and Mn^4+^ components shifted to lower binding energies of 641.25, 642.78, and 644.90 eV, respectively. These irradiation-dependent shifts indicate relative electron enrichment in Mn-related chemical environments and relative electron depletion in the Co–O framework. Together with the photocurrent, EIS, and PL results, these changes support light-induced charge redistribution in Mn5–Co_3_O_4_ and provide a spectroscopic basis for its prolonged carrier availability. Because binding-energy shifts alone do not directly resolve a complete microscopic transfer pathway or formal-valence conversion, they are interpreted as evidence of relative electronic redistribution rather than as proof of specific redox-state changes.

**Figure 6:**
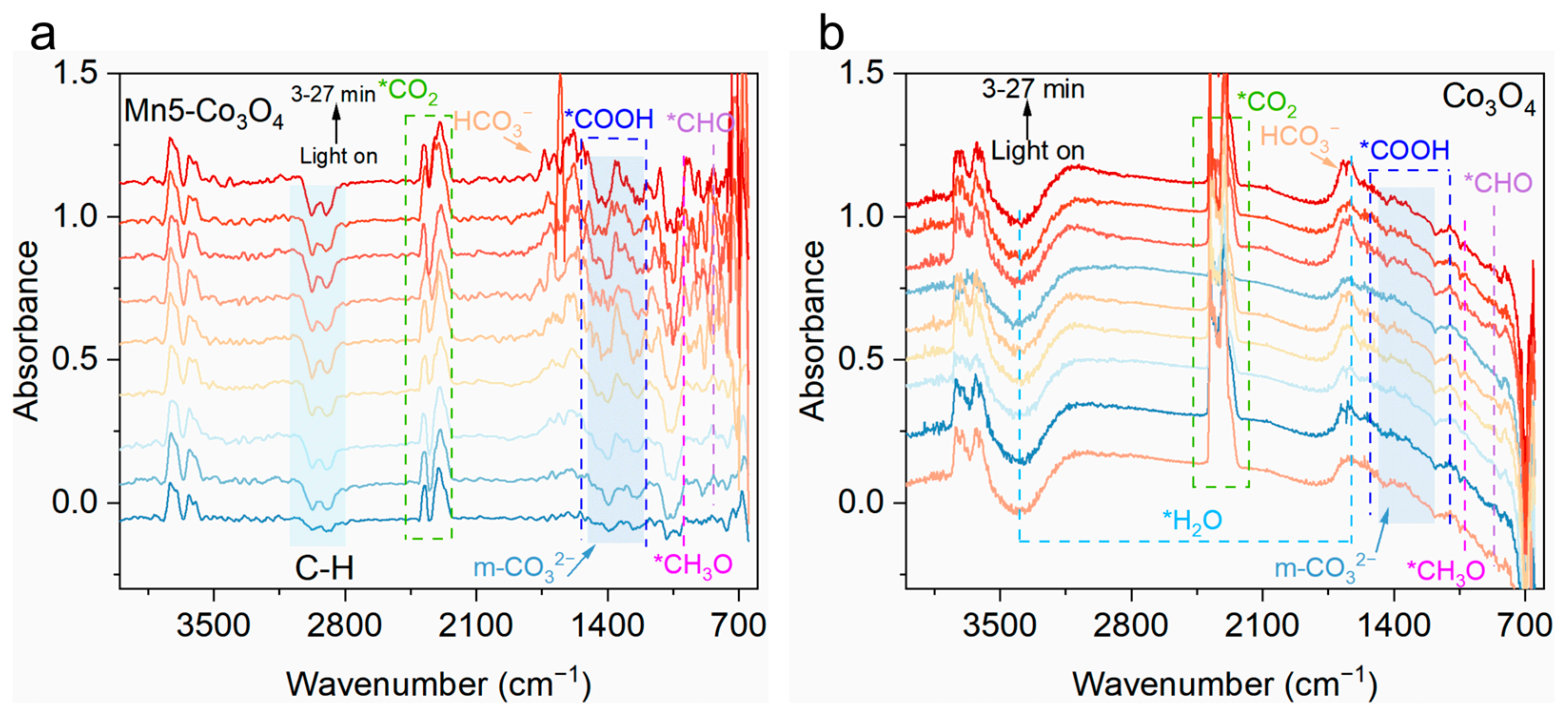
Time-resolved in situ FTIR spectra of (**a**) Mn5–Co_3_O_4_ and (**b**) Co_3_O_4_ collected under CO_2_/H_2_O and illumination.

Time-resolved in situ Fourier-transform infrared (FTIR) spectra were collected for Co_3_O_4_ and Mn5–Co_3_O_4_ under a CO_2_/H_2_O atmosphere to probe the effect of Mn doping on deep hydrogenation. As shown in Figure 6, both Co_3_O_4_ and Mn5–Co_3_O_4_ exhibited a characteristic feature at 2300–2400 cm^−1^ assigned to adsorbed CO_2_ molecules, together with an absorption band at 1670 cm^−1^ and a band in the 1330–1472 cm^−1^ region assigned to bicarbonate (HCO_3_^−^) and monodentate carbonate (m-CO_3_^2−^) species, respectively [56,57,58]. The coexistence of these configurations indicates co-adsorption of CO_2_ and H_2_O on both surfaces. During irradiation, additional bands emerged at 1524 and 1100 cm^−1^ on Mn5–Co_3_O_4_, assigned to the *COOH intermediate (where * denotes an adsorption site) formed from proton-coupled activation of adsorbed CO_2_ [59]. As the irradiation time increased from 3 to 27 min, two additional bands appeared at 1020 and 870 cm^−1^ and progressively intensified. These bands were tentatively assigned to *CH_3_O and *CHO species, respectively [23,60]. The sustained growth of these bands supports the continued hydrogenation of adsorbed carbon species from *COOH through *CHO and *CH_3_O toward CH_4_.

By contrast, pristine Co_3_O_4_ exhibited a distinct spectral evolution under identical conditions. Bands associated with *COOH were also detected, indicating that the initial protonation of adsorbed CO_2_ can occur on the undoped surface. However, the bands tentatively assigned to *CHO and *CH_3_O remained weak and increased only marginally over the same irradiation period. This limited accumulation of deeply hydrogenated intermediates is consistent with the lower CH_4_ production rate of pristine Co_3_O_4_ and its CH_4_/CO production-rate ratio of 0.67. The two catalysts showed comparable spectral signatures for CO_2_ adsorption and *COOH formation but markedly different evolution of *CHO and *CH_3_O. This comparison localizes the principal influence of Mn doping to the hydrogenation steps downstream of *COOH formation rather than to CO_2_ capture or initial activation. This interpretation is further supported by the carrier-dynamics results. Mn5–Co_3_O_4_ exhibited a longer average photoluminescence lifetime than pristine Co_3_O_4_, consistent with the prolonged availability of reductive charge carriers for successive proton-coupled electron-transfer events. Taken together, these results support a mechanistic sequence initiated by Mn incorporation and light-induced charge redistribution between Mn-related sites and the Co–O framework. The associated suppression of charge recombination and extension of carrier lifetime sustain the stepwise hydrogenation of *COOH through *CHO and *CH_3_O, thereby enhancing CH_4_ formation and increasing the CH_4_/CO ratio. Appropriate Mn doping therefore provides an effective strategy for coupling charge-carrier availability with the multistep hydrogenation required for photocatalytic CO_2_ methanation.

## 3. Materials and Methods

### 3.1. Materials

Cobalt nitrate hexahydrate (Co(NO_3_)_2_·6H_2_O, ≥99.9%, Adamas, Shanghai, China), manganese acetate tetrahydrate (Mn(CH_3_COO)_2_·4H_2_O, ≥99%, Adamas, Shanghai, China), cetyltrimethylammonium bromide (CTAB, ≥99%, Shanghai Aladdin Biochemical Technology Co., Ltd., Shanghai, China), 2-methylimidazole (2-MIM, ≥99%, Adamas, Shanghai, China), sodium sulfate (Na_2_SO_4_, ≥99%, Sinopharm Chemical Reagent Co., Ltd., Shanghai, China), potassium ferricyanide (K_3_[Fe(CN)_6_], ≥99%, Sinopharm Chemical Reagent Co., Ltd., Shanghai, China), potassium ferrocyanide (K_4_[Fe(CN)_6_], ≥99%, Sinopharm Chemical Reagent Co., Ltd., Shanghai, China) and ethanol (≥99.7%, Adamas, Shanghai, China) were used as received without further purification. High-purity CO_2_ (99.999%) was used as the reactant gas, and Ar (99.999%) was used as the carrier gas for gas-chromatographic analysis. Deionized water with a resistivity of 18.2 MΩ·cm was used throughout the experiments.

### 3.2. Preparation of Photocatalysts

The Mn-doped Co_3_O_4_ samples were prepared by precursor co-assembly followed by thermal conversion. Typically, 100 mg of Co(NO_3_)_2_·6H_2_O and 5 mg of Mn(CH_3_COO)_2_·4H_2_O were dissolved in 2 mL of deionized water containing 2 mg of CTAB. The resulting solution was rapidly added to 14 mL of deionized water containing 1.816 g of 2-MIM and vigorously stirred at room temperature for 20 min. The resulting CoMn precursor was collected by centrifugation, washed three times with ethanol, and dried under vacuum at 60 °C for 12 h. The dried precursor was heated in a muffle furnace to 320 °C at 5 °C min^−1^ and held at 320 °C in air for 2 h to obtain Mn5–Co_3_O_4_.

Three Mn precursor dosages were selected to establish an exploratory low-to-high series with a 1:2:4 progression while keeping the amount of the Co precursor and all other synthesis parameters constant. Specifically, 5, 10, or 20 mg of Mn(CH_3_COO)_2_·4H_2_O was combined with 100 mg of Co(NO_3_)_2_·6H_2_O, corresponding to nominal Mn/Co feed molar ratios of approximately 0.059, 0.119, and 0.237, respectively. This dosage range enabled systematic evaluation of the influence of the Mn precursor dosage on the catalyst structure, charge-carrier dynamics, and photocatalytic product distribution. The resulting samples were denoted Mn5–Co_3_O_4_, Mn10–Co_3_O_4_, and Mn20–Co_3_O_4_, where 5, 10, and 20 refer exclusively to the mass of Mn(CH_3_COO)_2_·4H_2_O used during synthesis, expressed in milligrams, rather than to the elemental Mn mass or the final Mn content of the catalysts. Pristine Co_3_O_4_ was prepared without the Mn precursor under otherwise identical conditions.

### 3.3. Characterization

Crystalline phases were analyzed by X-ray diffraction (XRD, D8 Advance, Bruker AXS GmbH, Karlsruhe, Germany) using Cu Kα radiation. Rietveld refinements of the pristine Co_3_O_4_ and Mn5–Co_3_O_4_ diffraction patterns were performed using GSAS-IIversion 5782 with a cubic spinel structural model in the Fd−3m space group. The corresponding crystallographic and atomic-site parameters are summarized in Table 1 and Table 2. The bulk Co and Mn contents were determined using an iCAP PRO X Duo ICP-OES instrument (Thermo Fisher Scientific, Waltham, MA, USA). Morphology, lattice fringes, and elemental distributions were examined by transmission electron microscopy (TEM), high-resolution TEM (HRTEM), and high-angle annular dark-field scanning TEM (HAADF-STEM) coupled with energy-dispersive X-ray spectroscopy (EDS) on a Talos F200i microscope (Thermo Scientific, Waltham, MA, USA) operated at 200 kV. Ultraviolet–visible diffuse reflectance spectra were recorded over 200–800 nm on a Lambda 950 spectrophotometer (PerkinElmer, Waltham, MA, USA) using BaSO_4_ as the reflectance standard, and the lower-energy apparent optical transition energies were estimated from the transformed Kubelka–Munk function versus photon energy using a direct-allowed Tauc construction. Steady-state photoluminescence (PL) and time-resolved photoluminescence (TRPL) spectra were recorded at room temperature on an FLS1000 spectrometer (Edinburgh Instruments Ltd., Livingston, UK) with an excitation wavelength of 375 nm, and identical excitation and acquisition conditions were applied to all samples. The TRPL decay curves were fitted with a biexponential function, and the average PL lifetime was calculated as τ_ave_ = (A_1_τ_1_^2^ + A_2_τ_2_^2^)/(A_1_τ_1_ + A_2_τ_2_), where A_i_ and τ_i_ denote the amplitude and lifetime of each decay component. In situ irradiated X-ray photoelectron spectroscopy (XPS) measurements were performed on an ESCALAB Xi^+^ spectrometer (Thermo Fisher Scientific, Waltham, MA, USA) using Al Kα radiation, with a 300 W Xe lamp used for irradiation. The near-surface atomic composition was estimated from the dark-state XPS survey spectrum using background-subtracted Co 2p, Mn 2p, and O 1s peak areas corrected with the relative sensitivity factors applied during XPS quantification. Adventitious carbon was excluded from the normalization.

### 3.4. Photoelectrochemical Measurements

Transient photocurrent responses, electrochemical impedance spectroscopy (EIS), and Mott–Schottky measurements were carried out on a CHI-660E (CH Instruments, Shanghai, China) electrochemical workstation in a standard three-electrode configuration. To prepare the working electrode, 5 mg of photocatalyst was ultrasonically dispersed in 400 μL of deionized water for 20 min, and 20 μL of the resulting suspension was drop-cast onto a 0.5 × 0.5 cm^2^ area of fluorine-doped tin oxide (FTO) glass and dried naturally. A platinum wire and an Ag/AgCl electrode in saturated KCl were used as the counter and reference electrodes, respectively, and the electrolyte contained 0.1 M Na_2_SO_4_ and 0.05 M K_3_[Fe(CN)_6_]/K_4_[Fe(CN)_6_]. A 300 W Xe lamp (MC-PF300B, Beijing CEAULIGHT Technology Co., Ltd., Beijing, China) was used as the light source. Transient photocurrent responses were recorded at an applied potential of 0 V versus Ag/AgCl under chopped illumination. EIS measurements were conducted in the dark at applied potentials of 0, 0.1, and 0.2 V versus Ag/AgCl over a frequency range of 10 Hz to 100 kHz, using an AC perturbation amplitude of 5 mV. The Nyquist plots presented in Figure 4b correspond to measurements performed at 0.1 V versus Ag/AgCl. The Mott–Schottky plots presented in Figure 2c were recorded at 1500 Hz. The flat-band potentials (*E*_fb_) obtained from the intercepts of the Mott–Schottky plots were converted to the normal hydrogen electrode (NHE) scale according to *E*_NHE_ = *E*_Ag/AgCl_ + 0.197 V, and the conduction band minimum of an n-type semiconductor was taken to be 0.1 V more negative than the flat-band potential. The valence-band maximum was then estimated by combining the conduction-band position with the corresponding lower-energy apparent optical transition energy.

### 3.5. Photocatalytic CO_2_ Reduction

Photocatalytic CO_2_ reduction was performed in a sealed 100 mL jacketed quartz reactor without any sacrificial agent. In each experiment, 20 mg of photocatalyst was uniformly dispersed at the bottom of the reactor and 2 mL of deionized water was introduced. The reactor was evacuated and refilled with high-purity CO_2_ (99.999%) three times to remove air and was then filled with CO_2_. The system was irradiated with the full spectrum of a 300 W Xe lamp, while a circulating cooling system maintained the reactor at 25 °C. The lamp intensity and lamp-to-reactor distance were kept constant in all comparative experiments. Gaseous products were analyzed on a GC9790 Plus gas chromatograph (Zhejiang Fuli Analytical Instruments Co., Ltd., Wenling, China) equipped with thermal-conductivity and flame-ionization detectors, using Ar as the carrier gas. The electron-based CH_4_ fraction among the measured carbon-containing products was calculated as 8rCH_4_/(8rCH_4_ + 2rCO) × 100%, where rCH_4_ and rCO denote the CH_4_ and CO production rates, respectively. The coefficients 8 and 2 correspond to the numbers of electrons required for CO_2_ reduction to CH_4_ and CO, respectively.

### 3.6. In Situ Fourier-Transform Infrared Spectroscopy

Time-resolved in situ Fourier-transform infrared (FTIR) spectra were acquired using an iS50 spectrometer (Thermo Fisher Scientific, Waltham, MA, USA) equipped with a mercury cadmium telluride detector and a diffuse-reflectance reaction cell. Spectra were collected at a resolution of 4 cm^−1^ using 32 accumulated scans. The photocatalyst was loaded into the in situ cell. A CO_2_ stream carrying H_2_O vapor was generated by bubbling high-purity CO_2_ through deionized water at room temperature and introduced for 30 min to establish adsorption equilibrium. A background spectrum was collected in the dark immediately before illumination. The reaction was then initiated under irradiation from a 300 W Xe lamp and spectra were collected at 3 min intervals. Identical gas-introduction, illumination and acquisition procedures were applied to Co_3_O_4_ and Mn5–Co_3_O_4_.

## 4. Conclusions

In summary, this work demonstrates that an appropriate Mn doping level in Co_3_O_4_ couples charge regulation with the sequential hydrogenation of CO_2_-derived intermediates, enabling sacrificial-agent-free CO_2_ photoreduction toward CH_4_ with H_2_O. The optimized Mn5–Co_3_O_4_ delivered a CH_4_ production rate 10.6-fold higher than that of pristine Co_3_O_4_, raised the CH_4_/CO molar ratio from 0.67 to 1.72, while showing more effective charge separation and an increase in the average PL lifetime from 9.95 to 23.17 ns. In situ irradiated XPS revealed that the Mn 2p signals shifted negatively under illumination, whereas the Co 2p and lattice O 1s signals shifted positively, indicating that photogenerated electrons accumulate at Mn sites while holes remain on the Co–O framework. Together with the enhanced *COOH, *CHO and *CH_3_O bands resolved by in situ FTIR, these results support a mechanism in which the light-induced charge redistribution prolongs carrier availability and thereby sustains the progressive hydrogenation of adsorbed carbon species. Previous studies of Mn-modified Co_3_O_4_ attributed enhanced CO_2_ methanation to adjacent Mn–Co redox sites and spin polarization, whereas atomic Mn incorporation under dilute CO_2_ and pure-water conditions preferentially promoted CO formation [23,39]. Relative to these reports, the systematic variation in Mn precursor dosage in the present study identified a shift from CO-dominant to CH_4_-dominant carbon-product formation and linked this shift to the measured carrier lifetime and the downstream hydrogenation of *COOH-derived intermediates. Beyond demonstrating the activity enhancement achieved by compositional modification, this work highlights the coordination between charge localization and surface hydrogenation as a design consideration for deep CO_2_ reduction.

## Figures and Tables

**Table 1:** ICP-OES-derived bulk cation composition of Mn5–Co_3_O_4_ and Rietveld-refinement parameters for Co_3_O_4_ and Mn5–Co_3_O_4_.

Parameter	Co_3_O_4_	Mn5–Co_3_O_4_
Crystal system	Cubic	Cubic
Space group	Fd−3m (No. 227)	Fd−3m (No. 227)
Bulk cation-normalized composition ^a^	Co_3_O_4_	Co_2.875_Mn_0.125_O_4_
Structural model	Co_3_O_4_-type spinel	Mn-incorporated Co_3_O_4_-type spinel
Mn site in the refinement model ^b^	—	Octahedral 16d (0.5, 0.5, 0.5)
a = b = c (Å)	8.0799 (7)	8.0847 (12)
α = β = γ (°)	90	90
Unit-cell volume (Å^3^)	527.50 (14)	528.44 (23)
Formula units per cell, Z	8	8
R_p_ (%)	1.72	1.80
R_wp_ (%)	2.15	2.25
χ^2^	1.01	1.02

^a^ For Mn5–Co_3_O_4_, the cation-normalized composition was derived from ICP-OES by normalizing the measured Co and Mn contents to three total metal cations under the Co_3_O_4_-type spinel convention. The oxygen stoichiometry was not independently determined. ^b^ In the Mn5–Co_3_O_4_ Rietveld model, Mn was placed together with Co at the octahedral 16d site, whereas the tetrahedral 8a site was occupied by Co. This structural model was used to assess the crystallographic Mn-site occupancy and not to determine the bulk Mn content.

**Table 2:** Atomic-site parameters of the Rietveld structural models.

Sample	Atom/Site	Wyckoff Site	Coordination	x	y	z	Site Constituent(s)	Uiso (Å^2^)
Co_3_O_4_	Co1	8a	Tetrahedral	0.12500	0.12500	0.12500	Co	0.0195
Co_3_O_4_	Co2	16d	Octahedral	0.50000	0.50000	0.50000	Co	0.0103
Co_3_O_4_	O1	32e	—	0.26020	0.26020	0.26020	O	0.0282
Mn5–Co_3_O_4_	Co1	8a	Tetrahedral	0.12500	0.12500	0.12500	Co	0.0280
Mn5–Co_3_O_4_	Co2/Mn	16d	Octahedral	0.50000	0.50000	0.50000	Co/Mn	0.0167
Mn5–Co_3_O_4_	O1	32e	—	0.26474	0.26474	0.26474	O	0.0168

## Data Availability

The data presented in this study are available within the article. The raw data are available from the corresponding author upon reasonable request.
